# Weathering Characteristics of Wood Plastic Composites Reinforced with Extracted or Delignified Wood Flour

**DOI:** 10.3390/ma9080610

**Published:** 2016-07-23

**Authors:** Yao Chen, Nicole M. Stark, Mandla A. Tshabalala, Jianmin Gao, Yongming Fan

**Affiliations:** 1 MOE Key Laboratory of Wooden Material Science and Application, Beijing Forestry University, Beijing 100083, China; gaojm@bjfu.edu.cn (J.G.); fanym@bjfu.edu.cn (Y.F.); 2 U.S. Department of Agriculture, Forest Service, Forest Products Laboratory, One Gifford Pinchot Drive, Madison, WI 53726-2398, USA; nstark@fs.fed.us (N.M.S.); mtshabalala@fs.fed.us (M.A.T.)

**Keywords:** WPCs, weathering, degradation, delignification, extractives

## Abstract

This study investigated weathering performance of an HDPE wood plastic composite reinforced with extracted or delignified wood flour (WF). The wood flour was pre-extracted with three different solvents, toluene/ethanol (TE), acetone/water (AW), and hot water (HW), or sodium chlorite/acetic acid. The spectral properties of the composites before and after artificial weathering under accelerated conditions were characterized by Fourier transform infrared (FTIR) spectroscopy, the surface color parameters were analyzed using colorimetry, and the mechanical properties were determined by a flexural test. Weathering of WPC resulted in a surface lightening and a decrease in wood index (wood/HDPE) and flexural strength. WPCs that were reinforced with delignified wood flour showed higher ΔL^*^ and ΔE^*^ values, together with lower MOE and MOR retention ratios upon weathering when compared to those with non-extracted control and extracted WF.

## 1. Introduction

In previous studies, we investigated the effect of extraction and delignification on the surface color, chemistry, and thermal properties of wood flour (WF) [1,2]. In a subsequent study, we reported on moisture and mechanical properties of WPCs that were made of extracted and delignified WF [3].

Currently, about two-thirds of WPCs are used in outdoor construction as materials for decking, siding, and roof tiles [4]. Outdoor uses can lead to deterioration of WPCs by abiotic agents such as moisture, sunlight, and temperature. Fading color and loss of mechanical properties of WPCs caused by outdoor weathering has become a great concern [5,6,7,8,9,10]. Several research groups have been working on characterizing and understanding changes that occur when WPCs weather [11,12,13,14,15,16,17]. Wood fiber swells after absorbing moisture and creates cracks in the plastic matrix which contribute to the loss of composite MOE and strength. Moisture also degrades the wood-plastic interface and decreases stress transfer efficiency from matrix to fiber [18,19]. UV radiation degrades the WPCs surface, further causing it to become cracked and flaky. Synergism between UV radiation and water also contribute to mechanical property losses of WPCs. The surface is eroded through water spray during weathering, providing new surfaces for degradation [20]. When wood-flour-filled high-density polyethylene (HDPE) composites were exposed to xenon-arc radiation either with or without water spray in an accelerated weathering apparatus, more lightening of the composite color occurred [21].

Although photodegradation of both polyethylene (PE) and wood have been extensively examined, little work has provided insight into the effect of lignin and extractives on the weathering characteristics of WPCs outdoors. Photodegradation of WPCs is a difficult problem, complicated by the fact that each component may degrade via a different mechanism. Increasing the wood content leads to decreased weathering performance of WPCs [21]. This is attributed to the hydrophilic nature of the wood fiber. The individual components of wood, cellulose, hemicellulose, lignin, and extractives are variously susceptible to photodegradation [22]. Research has shown that the weathering of wood is confined to the wood surface and involves photo-induced breakdown of lignin to water-soluble reaction products, which leads to the generation of chromophoric functional groups such as carbonyls, carboxylic acids, quinones, and hydroperoxy radicals [22]. When the wood undergoes photodegradation, the lignin degrades preferentially leaving a cellulose-rich surface. This increases surface wettability, causing WPCs to become more sensitive to moisture [23]. Wood extractives are also important agents with respect to weathering performance of wood. Removing extractives from wood flour (WF) before incorporation into WPCs is considered a good method to improve weathering performance [24]. Saputra et al. removed extractives from WF using three solvents including acetone/water, dioxane/water, or benzene/ethanol [25]. The extracted WF was combined with polypropylene to manufacture a WPC. Both flexural strength and stiffness improved when solvent extracted WF was used as reinforcement. In an effort to improve color stability through the removal of water soluble extractives, Stark and Mueller washed salt cedar (*Tamarix chiensis*) and pine WF in water before incorporation into a polyethylene matrix [21]. This decreased the water soluble extractive content from 12% to 3% for the salt cedar WF and from 6% to 3% for the pine WF. The WPCs then underwent accelerated weathering. There was no change in the color stability of the WPCs after weathering. Fabiyi et al. also modified WF to improve weathering performance [24]. Two techniques, acetone and acetic acid, were used to remove the extractives and lignin, respectively. The acetone extraction resulted in 3% extractive loss. The resultant wood fiber contained 1%–1.5% lignin after acetic acid extraction. WPCs manufactured from polyethylene and the modified wood fibers were then weathered. It was found that weathering discoloration of WPC reinforced with extracted wood flour was less pronounced compared with WPC reinforced with deliginified WF. Since the earlier studies used WF that was extracted with a single solvent, it was interesting to investigate whether WF that had been pre-extracted with multiple solvents or completely delignified would yield a different result. 

In the current work, three different solvents, toluene/ethanol (TE), acetone/water (AW), and hot water (HW) were used to remove extractives. Acidified sodium chlorite (ASC) was used for delignification. The surface chemistry and color changes of the WPCs after weathering were characterized by FTIR and colorimetry, and the mechanical properties were characterized by MOE measurements. 

## 2. Materials and Methods

### 2.1. Materials

The pine WF supplied by American Wood Fibers (AWF 4020; Schofield, WI, USA) was derived from mixed pine species. It was selected because it is the most common WF used in commercial WPC decking. Wood flour was sieved through a 40-mesh screen (0.425 mm) to remove the larger particles and through a 60-mesh screen (0.250 mm) to remove the fine particles. HDPE with a melt flow index of 33.0 g/10 min (according to ASTM D1238 [26] at load of 2.16 kg and temperature of 190 °C) and density of 0.951 g/cm^3^ (59.3 lb/ft^3^), was supplied by ExxonMobil (ExxonMobil Excorene HD-6733 HDPE; Houston, TX, USA).

### 2.2. Wood Flour Extractions

Three different solvent systems, toluene/ethanol (TE), acetone/water (AW), and hot-water (HW) were used to remove extractives from WF. TE solvent was prepared based on volume ratio (2:1) of toluene and ethanol. AW solvent was prepared based on volume ratio (9:1) of acetone and distilled water. Each extraction process was performed for 24 h in a Soxhlet apparatus (Corning Inc., New York, NY, USA) to ensure exhaustive removal of extractives. Delignification (LR) was accomplished by a method developed by Wise et al. with slight modifications [27]. A 100 g sample of air-dried WF was dispersed (under constant stirring) in 1 L of deionized water containing 30 g of NaClO_2_ (80%), 10 mL of acetic acid, and 100 μL of octan-2-ol (antifoaming agent, Acros Organics, Morris Plains, NJ, USA), and heated to 70 °C for 1 h. A further aliquot of 30 g of NaClO_2_ (80%) and 10 mL of acetic acid was added, and the reaction was continued for another 1 h. Addition of NaClO_2_ and acetic acid was repeated three more times for a total reaction time of 5 h before the mixture was allowed to cool to room temperature. The liquid layer was siphoned off, and the delignified WF was rinsed with 1 L aliquots of reverse-osmosis (RO) water until a neutral pH was attained. The wood flour slurry was filtered on a Buchner funnel lined with filter paper. The filter cake was rinsed with 1 L of RO water, followed by 500 mL of 95% ethanol, 500 mL of absolute ethanol, and 1 L of acetone. The filter cake was air-dried under suction for 30 min. All samples of WF, including the untreated WF were vacuum-dried before further experimentation. All WF was oven-dried at 120 °C for 24 h prior to composite processing.

### 2.3. HDPE Composites Manufacturing

Injection-molded WPC composite samples were manufactured in a micro-processing equipment (DSM Xplore, DSM Research, Geleen, The Netherlands). This system consists of a 15 mL conical, twin-screw compounder and a 12 mL injection molder, which allows for processing in small batch sizes, during which time the process conditions can be monitored and controlled. The production temperature was 180 °C. After compounding via extrusion, the extrudate was collected and injection-molded. An ASTM flexural property die was used with mold cavity dimensions of 3 × 12 × 120 mm^3^ [28]. All WPC samples were made of 50% by weight WF and 50% by weight HDPE, with the treatment of WF changing.

### 2.4. HDPE Composites Weathering

HDPE composites were placed in a xenon arc-type light exposure apparatus operated according to ASTM D2565 (Ci5000, Atlas Weathering, Chicago, IL, USA) [29]. Samples were mounted on a drum that rotates around a xenon arc bulb at 1 rpm. Each 2 h weathering cycle consisted of 108 min of UV exposure and 12 min of simultaneous water spray and UV exposure. The samples were exposed for 2000 h. The average irradiance was 0.70 W/m^2^ at 340 nm wavelength. The test lamp was calibrated every 400 h of irradiation and the inner filter of the test lamp was changed simultaneously.

### 2.5. Color Measurement

The surface color of weathered and non-weathered WPC composite samples was measured with a Minolta CR-400 Chroma Meter (Konica Minolta Sensing, Inc., Osaka, Japan). The CIELAB color system was used to determine the surface color in L^*^, a^*^, b^*^ coordinates based on a C light source. L^*^ represents the lightness coordinate and varies from 100 (white) to 0 (dark), a^*^ represents the red (+a^*^) to green (−a^*^) coordinate, and b^*^ represents the yellow (+b^*^) to blue (−b^*^) coordinate. The color difference was calculated as outlined in ASTM D2244 [30] according to the following equation:

(1) $$\Delta E=\sqrt{\Delta L^{*2}+\Delta a^{*2}+\Delta b^{*2}}$$

where, ΔL^*^, Δa^*^, and Δb^*^ represents the difference between the initial and final values of L^*^, a^*^, and b^*^, respectively. An increase in L^*^ means the sample is lightening. A positive Δa^*^ signifies a color shift toward red, and a negative Δa^*^ signifies a color shift toward green. A positive Δb^*^ signifies a shift toward yellow, and a negative Δb^*^ signifies a shift toward blue. It should be noted that ΔE^*^ represents the magnitude of the color difference, but does not indicate the direction of the color difference. The surface color for five replicates was measured at five locations on each composite sample.

### 2.6. Fourier Transform Infrared Spectroscopy

The surface chemistry of the weathered and non-weathered WPCs was characterized by attenuated total reflectance (ATR) Fourier transform infrared spectroscopy (FTIR) using an ATR module, iz10 module, and iN10 scope (Thermo Scientific Verona, Madison, WI, USA). Each spectrum was taken as an average of 64 scans at a resolution of 4 cm^−1^, and recorded in absorbance units from 4000 to 650 cm^−1^. The surfaces of the samples analyzed were in contact with a ZnSe crystal with a 45° angle of incidence. The peaks were analyzed without smoothing the data. Net peak heights were determined by subtracting the height of the baseline immediately before the peak from the total peak height. The data are presented in the form of indices and concentrations of the functional groups of interest.

The changes of wood index (WI) and crystallinity of HDPE after weathering are the main indicators of the degree of degradation upon weathering. The changes of wood index were calculated using the following equation:

(2) $$WI={\left(\frac{I_{1030}}{I_{2912}}\right)}_{before}-{\left(\frac{I_{1030}}{I_{2912}}\right)}_{after}$$

where *I* represents peak intensity. The peak intensities were normalized using the peak at 2912 cm^−1^, which corresponds to alkane CH stretching and vibrations of the methylene groups (–CH_2_–). This peak was chosen as a reference peak because it changed the least during weathering. The absorbance at 1030 cm^−1^ is C–O in cellulose and primary alcohols of lignin.

Crystallinity of HDPE after weathering was determined using the method described by Zerbi et al. [31]. The doublet peaks observed at 730*–*720 cm^−1^ correspond to polyethylene crystalline content (730 cm^−1^) and amorphous content (720 cm^−1^). The percentage of the crystalline content, X, can be calculated using Equation (3):

(3) $$X=100-\frac{(1-I_{a}/I_{b})/1.233}{1+I_{a}/I_{b}}(100)$$

where, *I_a_* and *I_b_* represent the bands intensity at 730 cm^−1^ and 720 cm^−1^, respectively [32].

### 2.7. Mechanical Property Testing

The mechanical properties of each HDPE composite before and after weathering were determined. Samples were conditioned at 22 °C and 63% relative humidity (RH) for 30 days before testing, to ensure the same conditioning for samples before and after weathering. Flexural tests were carried out according to ASTM D790 (ASTM 8.01) [28] on an Instron universal testing machine (Canton, MA, USA). The three-point loading system was utilized with a crosshead speed of 1.3 mm/min. The exposed surface was placed away from the center load to place the exposed surface in tension to represent a worst-case scenario. Five replicate specimens were tested for each formulation. The flexural strength (MOR) and tangent modulus of elasticity (MOE) were calculated according to ASTM D790 (ASTM 8.01) [28]. The effect of weathering on the mechanical properties of the composites were evaluated by calculating the retention ratios. The MOR and MOE retention ratios were calculated using to Equations (4) and (5).

MOR_ret ratio_ = 100 × (MOR_after_/MOR_before_) (4)

MOE_ret ratio_ = 100 × (MOE_after_/MOE_before_) (5)

where MOR_before_, MOE_before_, MOR_after_, and MOR_after_ represent the MOR and MOE before and after weathering.

### 2.8. Scanning Electron Microscopy

Composite surfaces were sputtered with gold and analyzed with a scanning electron microscope (SEM) (JSM-840, JEOL USA, Inc., Peabody, MA, USA) at a working distance of approximately 25 mm, voltage of 15 kV, and a probe current of 1109 amps.

### 2.9. Statistical Analysis

An analysis of variance (ANOVA) was conducted to evaluate the effect of extraction or delignification on the mechanical properties of WPCs. Design Expert 7.0.0 software by Stat-Ease (Minneapolis, MN, USA) was used to design the experiment and analyze the data. The derived equations are reported in terms of actual factors.

## 3. Results and Discussion

### 3.1. Fourier Transform Infrared Spectroscopy

**Figure 1:**
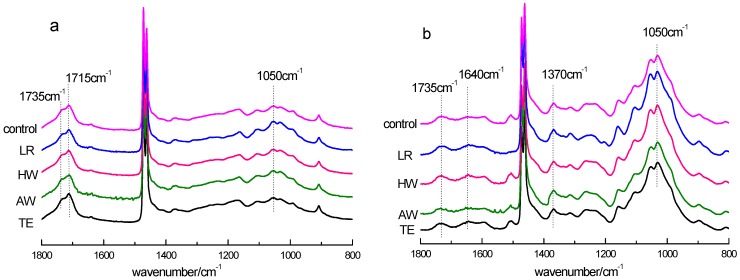
FTIR spectra of WPCs before and after weathering (**a**) after weathering; (**b**) before weathering.

FTIR spectra of WPCs produced with solvent extracted or delignified WF before and after weathering are presented in Figure 1. The peaks at 1050 cm^−1^, attributed to the linkage between the sugar units, decreased drastically upon weathering. It suggests the degradation of carbohydrates, especially the degradation of cellulose. However, the intensity of the band at 1750–1700 cm^−1^, which was assigned to the carbonyl groups (carboxylic acids at 1715 cm^−1^ and esters at 1735 cm^−1^), increased intensely. This implies that oxidation and photo-degradation of cellulose occurred upon weathering. It also means that the composites were vulnerable to further degradation because the carbonyl groups are photo-labile. A significant decrease in the intensity of peaks at 1033 cm^−1^ (C–O in cellulose and primary alcohols of lignin) occurred on the surface of WPCs after exposure. It suggested that the degradation of wood occurred intensely upon weathering. The intensity at 1640–1500 cm^−1^ decreased after weathering, which suggested the saturated conjugated structures, such as the carbonyls and the aromatic structures of lignin, are degraded through photo oxidation during the weathering process. This will be a contributor to the discoloration of the samples as discussed later.

**Figure 2:**
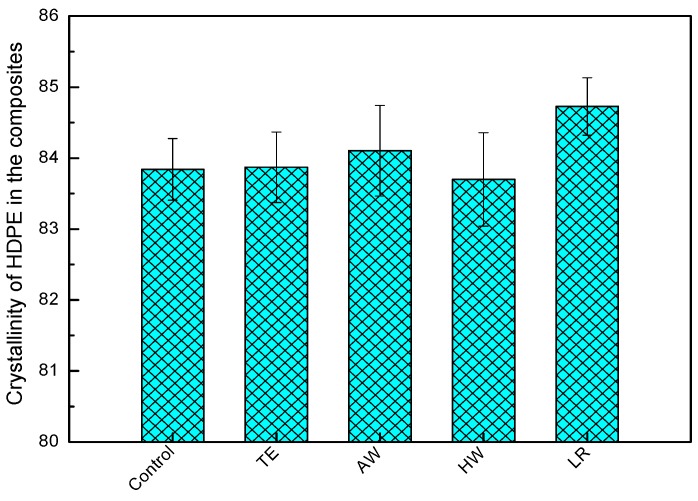
Crystallinity of HDPE in the samples after weathering.

The structural changes in the matrix after weathering were investigated by following crystallinity changes of HDPE in the composite samples (Figure 2). There was very slight difference in the crystallinity of HDPE when blended with TE extracted WF after weathering. The crystallinity of HDPE increased when blended with AW extracted WF, while decreased when blended with HW extracted WF. However, higher crystallinity of HDPE can be observed when blended with WF treated with delignification. It is reasonable to assume that the presence of lignin damages the crystallinity of HDPE upon weathering. It can be explained that lignin absorbs UV light producing free radicals, which causes further oxidation reactions [32]. A small amount of oxidation products in polyethylene can cause great damage to the tie molecules, resulting in a breakdown of crystallization [33]. Tie molecules are polymer molecules that form a part of a folded crystalline region and extend through an amorphous region into another crystalline region.

**Figure 3:**
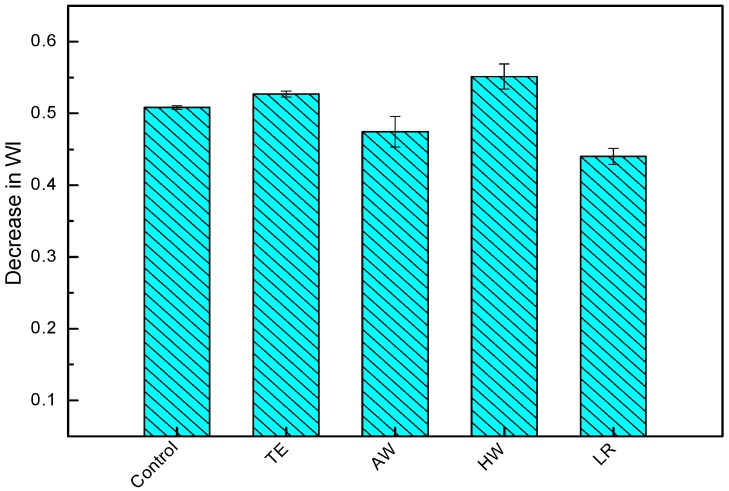
Decreased WI of the samples after weathering.

It can be seen from Figure 3, wood loss from WPC composites produced from delignified WF was slightly lower when compared to non-extracted control and extracted WF based composites. It is believed that water spray cycles during weathering washed away the loose wood components, and the presence of micro-cracks provided a channel for their fast removal on the composite surface. Wood losses were lower for the delignified and AW extracted wood flour filled WPCs. It was evident that lignin is more sensitive to photo-degradation than holocellulose (cellulose and hemicelluloses).

### 3.2. Color Parameter Changes

**Figure 4:**
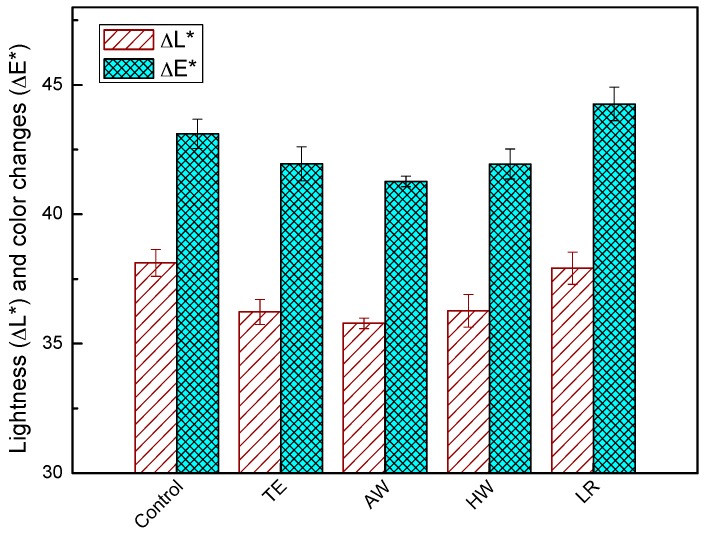
The lightness (ΔL^*^) and total color changes (ΔE^*^) of the WPCs after weathering.

Changes of lightness (ΔL^*^) and color (ΔE^*^) of WPCs made from extracted and delignified WF after weathering are shown in Figure 4.

The L^*^ values increased rapidly after weathering. The increase in lightness suggests degradation of substances that reflect yellowish and reddish light, resulting in a bleaching effect of the composite samples. The reason for color change is believed to be photo degradation of structures in wood components, especially conjugated structures, which degrade under UV radiation and water spray. Compared with the composite made from untreated control, the WPCs produced from TE, AW, or HW extracted WF exhibited lower ΔL^*^ and ΔE^*^ values, while those made from delignified WF showed no significant difference in ΔL^*^ and ΔE^*^ values. It can be inferred that degradation of extractives contributed to the color changes of WPCs to some extent upon xenon-arc accelerated weathering. There is no significant difference in ΔL^*^ and total color change ΔE^*^ of weathered WPC made from WF extracted with different solvents. This is reasonable since the structures in wood components are likely to photo-degrade unselectively. This is consistent with results from the analysis of FTIR spectra. As observed from the FTIR spectra (Figure 1), the absorption peaks attributed to cellulose (1000–1050 cm^−1^) and lignin (1505 cm^−1^ and 1595 cm^−1^) as well as other conjugated structures (1500–1640 cm^−1^) degraded significantly after weathering. 

### 3.3. Flexural Properties of Modified Wood Based WPC

**Figure 5:**
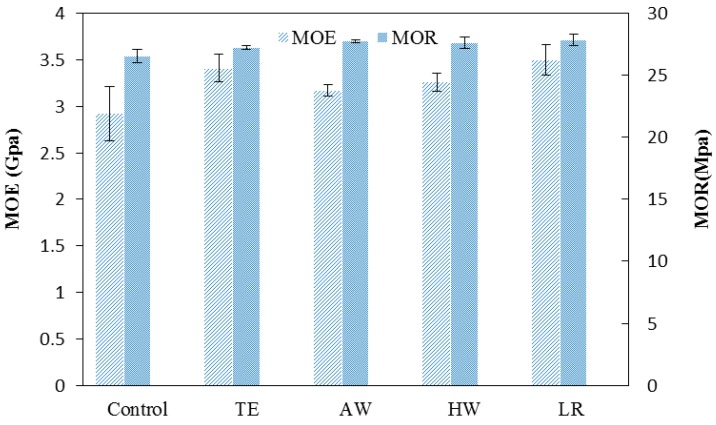
The MOE and MOR of the WPCs reinforced with extracted or delignified WF.

The flexural modulus of the rupture (MOR) and modulus of elasticity (MOE) of the WPC composite samples produced either from extracted or delignified WF was higher, on average, than those made from non-extracted WF (Figure 5). The samples that filled with delignified WF presented even greater MOE values than those with extracted WF. Better mechanical properties of WPCs prepared with extracted WF were attributed to the removal of polar extractives through ethanol, acetone/water, and hot water extraction. The compatibility between HDPE matrix and wood filler can be enhanced with decreased hydrophilicity of the WF. It has also been reported that the interfacial shear strength between the HDPE matrix and extracted wood filler improved after extraction [25].

**Figure 6:**
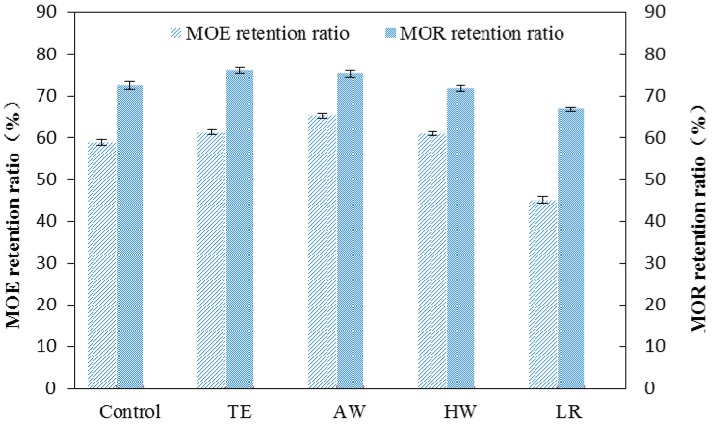
The MOE and MOR retention ratios of the WPCs reinforced with extracted or delignified WF.

The effects of accelerated UV weathering on flexural MOR and MOE of the composites reinforced with extracted and delignified WF are shown in Figure 6. Both MOR and MOE values decreased after weathering due to UV radiation and water spray. The MOE retention ratio of all the composites displayed the similar trend to that of MOR retention ratio. As HDPE is exposed to UV radiation, chain scission occurs. Additionally, exposure to water degrades mechanical properties of WPCs mainly due to swelling of the wood particles. The swelling particles cause microcracks in the matrix, causing a decrease in flexural strength, and reducing efficiency of stress transfer from fiber to the matrix, causing a decrease in strength. It is reported that WF/HDPE composites retained only 45% pre-exposure MOE after 4000 h of weathering [34]. In our experiment, the composites reinforced with extracted WF retained over 60% pre-exposure MOE. The WPCs made from TE or AW extracted WF retained over 70% MOR values after weathering. Compared with the composites made from extracted WF, the WPCs produced from delignified WF showed lower MOE and MOR retention ratios. After exposure, the MOR retention ratio of the composites were rated as TE > AW > Control > HW > LR. It is reasonable to assume that lignin contributes to the flexural strength of the composites upon weathering. Lignin has been found to play a major role in protecting the cellulose/hemicellulose from harsh environmental conditions such as water [35].

### 3.4. Surface Properties Analysis

**Figure 7:**
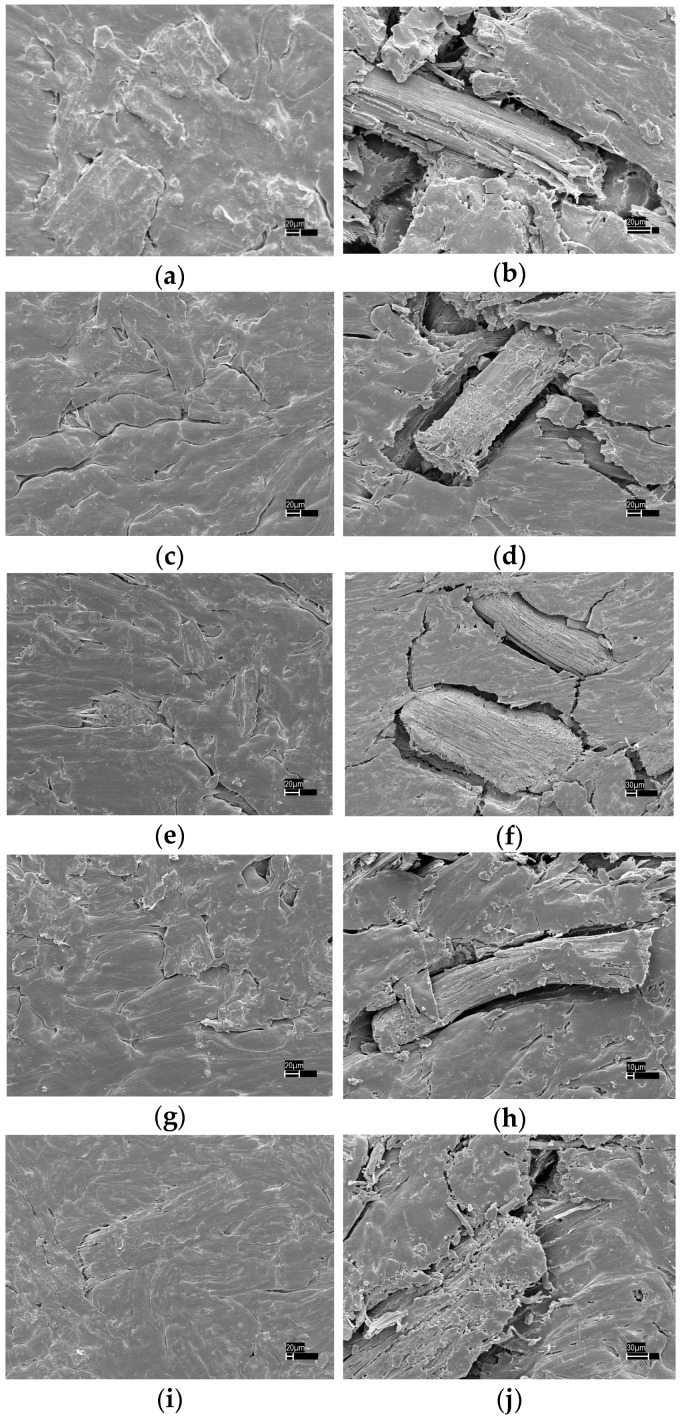
Morphology of the WPCs before and after weathering. (**a**) Control before weathering; (**b**) Control after weathering; (**c**) TE before weathering; (**d**) TE after weathering; (**e**) AW before weathering; (**f**) AW after weathering; (**g**) HW before weathering; (**h**) HW after weathering; (**i**) LR before weathering; (**j**) LR after weathering.

The surface morphology comparison of the samples before and after weathering is shown in Figure 7. The cracking between the polymer matrix and WF can be observed on the surface and at the interfacial region after weathering. In addition, wood particles protruded from the composite surface. It is likely that ultraviolet radiation aggravates photodegradation of HDPE causing disconnection in the wood and HDPE interface matrix. The protrusion of wood particles was likely a result of the wood particles swelling and shrinking aided by moisture absorption into hydrophilic wood fibers upon weathering. It is believed that PE undergoes photo-oxidation during exposure to UV, which results in increased carbonyl groups and decreased molecular weight of the fillers [32]. The reduction of molecular weight and crystallinity leads to the embrittlement of PE matrix. It can be seen from Figure 7 that the fractured surface of the control composites displayed holes, fibre pull-out, and matrix cracking after weathering, implying poor WF/HDPE adhesion. These phenomena were more pronounced in composites reinforced with delignified WF. In this situation, stress could not be transferred effectively in the composite matrix and in turn led to lower flexural properties. This was in accordance with the above flexural properties results.

### 3.5. Statistical Analysis

The results of variance analysis of factorial experimental design are given in Table 1. The main factor was selected for analysis in response to the decreased rates of MOE with interaction effects counted as residual. The designed model is found to be significant. The *F*-value for this model is 11.06 and the values of “Prob > F” less than 0.0500. It illustrates that factor LR is a significant term. This suggests that delignification has a greater impact on MOE reduction during weathering than extractive removal. It suggests that lignin can act as a degradation retardant due to its radical stabilizing effect, which reduces free radical degradation of the matrix when exposed to UV light.

## 4. Conclusions

In this study, the effect of weathering on WPC color, chemical composition, and flexural properties was examined. Weathering of WPC resulted in a surface lightening. WPC reinforced with TE, AW, or HW extracted WF exhibited less total color change and lightening when compared to non-extracted control after weathering. It seems that absence of extractives has a positive effect on reducing color changes of WPCs upon xenon-arc accelerated weathering. The unsaturated conjugated structures are changed through photo oxidation, which contribute to discoloration of the samples. The wood content of weathered WPC surfaces was decreased. Wood loss from WPC composites produced from delignified WF was slightly lower when compared to non-extracted control and extracted WF based WPC. The flexural strength of WPC samples produced with extracted wood flour were higher than those with untreated wood flour due to the removal of extractives. The samples filled with delignified wood flour showed lower flexural strength after weathering. WPCs are obviously damaged on the surface and at the interfacial region when exposed to UV light. Delignification has a greater effect on flexural strength reduction than extractives removed during weathering.

## Figures and Tables

**Table 1:** ANOVA of MOE reduction rate.

Source	Sum of Squares	df	Mean Square	F Value	*p*-Value Prob > F	Significant
Model	531.66	4	132.91	11.05	0.0008	significant
TE	7.07	1	7.07	0.58	0.4591	–
AW	31.02	1	31.02	2.57	0.1366	–
HW	2.98	1	2.98	0.24	0.6278	–
LR	490.59	1	490.59	40.81	< 0.0001	–
Residual	132.21	11	12.01	–	–	–
Corrected Total	663.87	15	–	–	–	–
